# Supplementary material for: All-trans Retinoic Acids Synergistically and Beneficially Affect In Vitro Glaucomatous Trabecular Meshwork (TM) Models Using 2D and 3D Cell Cultures of Human TM Cells
Source: Int J Mol Sci. 2022 Aug 31;23(17):9912. doi: 10.3390/ijms23179912 (PMC9456377; doi:10.3390/ijms23179912)
Supplement: Supplementary file 1 [file ijms-23-09912-s001.zip › ijms-1874463-supplementary.pdf]

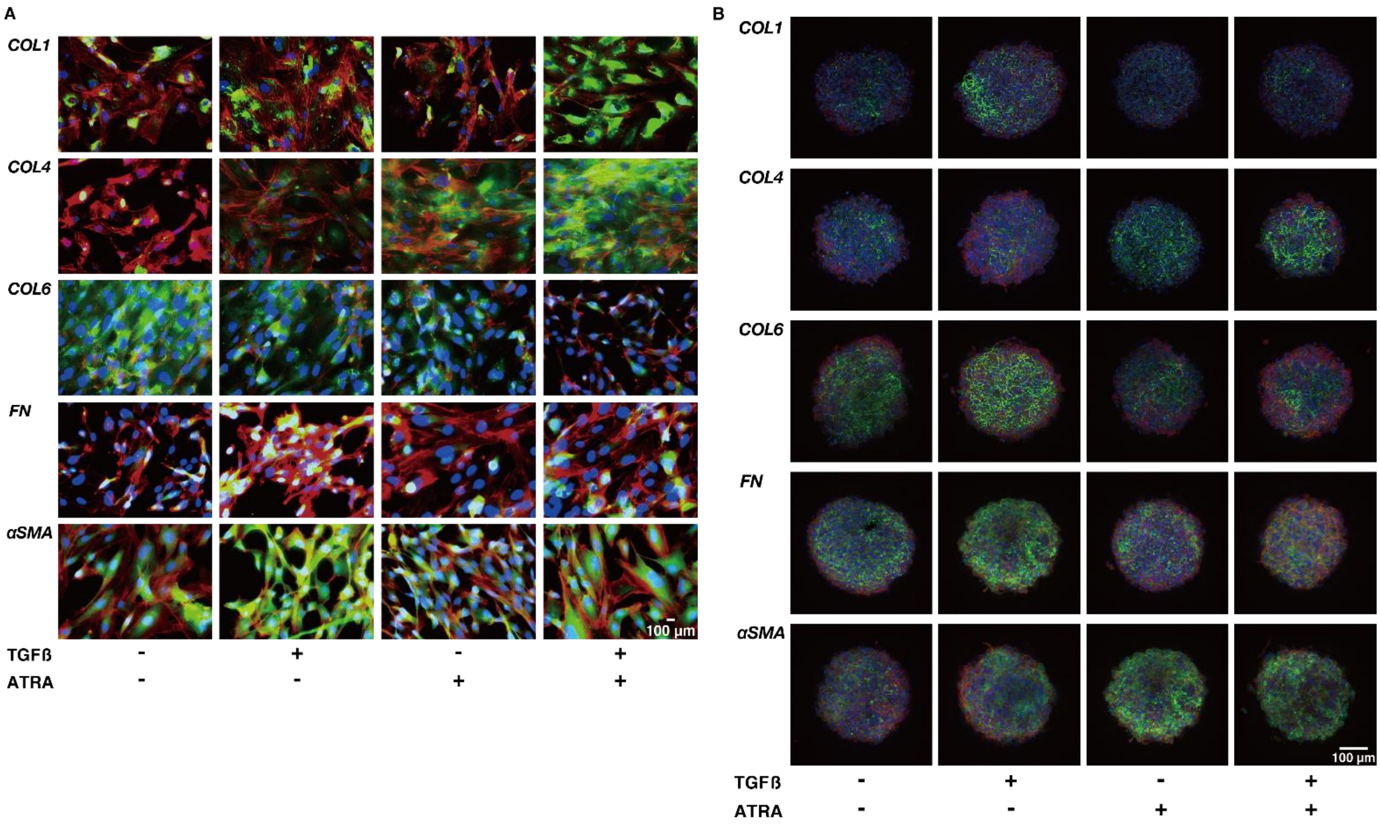

Supplemental Figure S1. Effects of ATRA on the immunolabeling of ECM proteins of the TGF-β2 untreated or treated 2D and 3D HTM cells.

2D and 3D HTM cells untreated and treated with TGF-β2 (TGFβ, 5 ng/ml) were subjected to immunolabeling for *COL 1*, *COL 4*, *COL 6*, *FN* and *αSMA* in the presence or absence of 10 μM ATRA. Experiments were repeated in duplicate (n=5 in each, total n=10). Representative merged images of ECM staining (green) with DAPI (blue) and Phalloidin (red) are shown in panels A (2D) and B (3D).

Supplemental Table S1 Sequences of primers of qPCR

		Sequence	Exon Location	RefSeq Number	Product Length (bp)
human RPLP0[*1]	Probe	5'-/56-FAM/CCCTGTCTT/ZEN/CCCTGGGCATCAC/3IABkFQ/-3'			
	Primer2	5'-TCGTCTTTAAACCCTGCGTG-3'	2-3	NM_001002	143
	Primer1	5'-TGTCTGCTCCCACAATGAAAC-3'			
human COL1A1[*1]	Probe	5'-/56-FAM/TCGAGGGCC/ZEN/AAGACGAAGACATC/3IABkFQ/-3'			
	Primer2	5'-GACATGTTCAGCTTTGTGGAC-3'	1-2	NM_000088	115
	Primer1	5'-TTCTGTACGCAGGTGATTGG-3'			
human COL4A1[*1]	Probe	5′-/56-FAM/TCATACAGA/ZEN/CTTGGCAGCGGCT/3IABkFQ/-3′	51-52		
	Primer2	5′-AGAGAGGAGCGAGATGTTCA-3′		NM_001845	142
	Primer1	5′-TGAGTCAGGCTTCATTATGTTCT-3′			
human COL6A1[*1]	Probe	5′-/56-FAM/CAGGTTTCG/ZEN/GTCACAGCGGTAGT/3IABkFQ/-3′			
	Primer2	5'-CCTCGTGGACAAAGTCAAGT-3'	2-3	NM_001848	114
	Primer1	5'-GTGAGGCCTTGGATGATCTC-3'			
human FN1[*1]	Probe	5′-/56-FAM/TACAGCTTA/ZEN/TTCTCCCTCGCCCAG/3IABkFQ/-3′			
	Primer2	5'-CGTCCTAAAGACTCCATGATCTG-3'	3-4	NM_212482	129
	Primer1	5'-ACCAATCTTGTAGGACTGACC-3'			
human αSMA[*1]	Probe	5'-/56-FAM/AGACCCTGT/ZEN/TCCAGCCATCCTTC/3IABkFQ/-3'			
	Primer2	5'-AGAGTTACGAGTTGCCTGATG-3'	8-9	NM_001613	105
	Primer1	5'-CTGTTGTAGGTGGTTTCATGGA-3'			
human TIMP1[*1]	Probe	5′-/56-FAM/TCAACCAGA/ZEN/CCACCTTATACCAGCG/3IABkFQ/-3′			
	Primer2	5′-CCTTCTGCAATTCCGACCT-3′	2-4	NM_003254	121
	Primer1	5′-GCTTGGAACCCTTTATACATCTTG-3′			
human TIMP2[*1]	Probe	5′-/56-FAM/TCTCATTGC/ZEN/AGGAAAGGCCGAGG/3IABkFQ/-3′			
	Primer2	5′-GACGTTGGAGGAAAGAAGGA-3′	3-4	NM_003255	133
	Primer1	5′-TGTGGTTCAGGCTCTTCTTC-3′			
human TIMP3[*1]	Probe	5′-/56-FAM/CCTCCTTTA/ZEN/CCAGCTTCTTCCCCAC/3IABkFQ/-3′			
	Primer2	5′-CCTTCTGCAACTCCGACATC-3′	1-3	NM_000362	112
	Primer1	5′-CGGTACATCTTCATCTGCTTGA-3′			
human TIMP4[*1]	Probe	5′-/56-FAM/ACTGAGGAC/ZEN/CTGACCAGTCAAGAGA/3IABkFQ/-3′			
	Primer2	5′-GGTTTGAGAAAGTCAAGGATGTTC-3′	3-4	NM_003256	149
	Primer1	5′-GTTGCACAGATGGATGAAGAC-3′			
human MMP2[*1]	Probe	5′-/56-FAM/TTCTGTCCC/ZEN/CATGAAGCCCTGTTC/3IABkFQ/-3′			
	Primer2	5′-TCCACCACCTACAACTTTGAG-3′	6-7	NM_004530	140
	Primer1	5′-GTGCAGCTGTCATAGGATGT-3′			
human MMP9[*1]	Probe	5′-/56-FAM/CCAGGAGGA/ZEN/AAGGCGTGTGC/3IABkFQ/-3			
	Primer2	5′-ACATCGTCATCCAGTTTGGTG-3′	3-4	NM_004994	123
	Primer1	5′-CGTCGAAATGGGCGTCT-3′			
human MMP14[*1]	Probe	5′-/56-FAM/TTGTTCCTC/ZEN/AAAGTGCCTGTTTGCTC/3IABkFQ/-3			
	Primer2	5′-TTCGCCGACTAAGCAGAAG-3′	1-1	NM_004995	114
	Primer1	5′-CTTGAATTCCTAGACCGCTGT-3′			
human ZO1[*1]	Probe	5'--/56-FAM/ACTGAATTA/ZEN/CCTTCACCATGTGCTCCC/3lABkFQ/-3'			
	Primer2	5'-CGCGTCTCTCCACATACATTC-3'	24-25	NM_ 175610	117
	Primer1	5'-GCTGGCTTATTCTGAGATGGA-3'			
human CLDN11[*1]	Probe	5'--/56-FAM/TGACTGCCT/ZEN/GCTTTGTGCTACGT/3lABkFQ/-3'			
	Primer2	5'-CATGGATTTCAGAACCTGCATT-3'	2-3	NM_ 001185056	129
	Primer1	5'-GGAAGAACAGTCAGCAGCA-3'			
human Grp78[*2]	Forward	5'-CATCACGCCGTCCTATGTCG-3'		NM_005347	215
	Reverse	5'-CGTCAAAGACCGTGTTCTCG-3'			
human GRP94[*2]	Forward	5'-CTGGGACTGGGAACTTATGAATG-3'		NM_003299	152
	Reverse	5'-TCCATATTCGTCAAACAGACCAC-3'			
human XBP[*2]	Forward	5'-AGTAGCAGCTCAGACTGCCA-3'		NM_005080	313
	Reverse	5'-CCTGGTTCTCAACTACAAGGC-3'			
human sXBP[*2]	Forward	5'-GGTCTGCTGAGTCCGCAGCAGG-3'		AB076384	97
	Reverse	5'-GGGCTTGGTATATATGTGG-3'			
human CHOP[*2]	Forward	5'-GGAGAACCAGGAAACGGAAAC-3'		NM_004083	69
	Reverse	5'-TCTCCTTCATGCGCTGCTTT-3'			

*1 Taqman probes (IDT, Coralville, IA, USA). *2 SYBR probes (IDT, Coralville, IA, USA).
